# Supplementary material for: Soft magnetic microrobots with remote sensing and communication capabilities
Source: Nat Commun. 2025 Nov 25;16:10489. doi: 10.1038/s41467-025-65459-8 (PMC12647796; doi:10.1038/s41467-025-65459-8)
Supplement: Supplementary file 1 — Supplementary information [file 41467_2025_65459_MOESM1_ESM.pdf]

Supplementary Information for

Soft Magnetic Microrobots with Remote Sensing and Communication Capabilities

Quan Gao,[1]† Minsoo Kim,[1]*† Denis von Arx,[1] Elric Zhang,[1] Xinzhi Zhang,[2] Hao Ye,[1] Christian Vogt,[3] Claas Ehmke,[1] Dianne Corsino,[4] Federica Catania,[4] Niko Münzenrieder,[4] Michele Magno,[3] Giuseppe Cantarella,[5, 6] Bradley J. Nelson,[1] Salvador Pané[1]

[1] Multi-Scale Robotics Lab, Institute of Robotics and Intelligent Systems, ETH Zurich, Zurich, Switzerland

[2] Institute of Electromagnetic Fields, ETH Zurich, Zurich, Switzerland

[3] Center for Project-Based Learning, ETH Zurich, Zurich, Switzerland

[4] Faculty of Engineering, Free University of Bozen-Bolzano, Bozen-Bolzano, Italy

[5] Department of Physics, Informatics and Mathematics, University of Modena and Reggio Emilia, Modena, Italy

[6] Istituto Nanoscienze CNR, Centro S3, 41125 Modena, Italy

*Corresponding author. Email: minkim@ethz.ch

†These authors contributed equally to this work.

This PDF file includes:

- Supplementary Figures:

Supplementary Fig. 1. Mechanism of helical structure formation via the interplay of hydrogel swelling and anisotropic supporting layer.

Supplementary Fig. 2. Alignment marker and spacer in mask design for precise layered structure fabrication.

Supplementary Fig. 3. Capability of using various electronics designs in integration.

Supplementary Fig. 4. Layer configuration integrating flexible electronics and soft microrobots.

Supplementary Fig. 5. Effect of the IONP concentration ratio on helical shape formation.

Supplementary Fig. 6. Magnetic navigation setup using three pairs of Helmholtz coils.

Supplementary Fig. 7. Navigation of planar microrobots using a magnetic gradient field.

Supplementary Fig. 8. Layout designs and both sides optical images of the microrobot in planar form, shown in various dimensions.

Supplementary Fig. 9. Shape reconfiguration repeatability test.

Supplementary Fig. 10. Experimental setup for evaluating shape detection performance through the radio communication.

Supplementary Fig. 11. Negligible effect of IONPs and hydrogel on radio communication.

Supplementary Fig. 12. Collective effect of microrobots with different dimensions on radio communication.

Supplementary Fig. 13. Simulation of the medium effect on radio communication.

Supplementary Fig. 14. Effect of the sample holder on radio communication.

Supplementary Fig. 15. Localization setup and shielded experimental setup.

Supplementary Fig. 16. Time for shape deformation from helical to planar as a function of temperature.

Supplementary Fig. 17. Resonant frequency of the new spiral antenna in air.

Supplementary Fig. 18. Effect of microrobot distribution density on RF communication using COMSOL simulation.

Supplementary Fig. 19. Experimental comparison of microrobots' RF signal response at the resonant frequency in PBS.

Supplementary Fig. 20. Experimental comparison of seven microrobots' RF signal response at two different depths.

Supplementary Fig. 21. Biocompatibility of microrobots.

Supplementary Fig. 22. Active layer fabrication setup.

Supplementary Fig. 23. Fabrication and integration workflow chart.

Supplementary Fig. 24. Stability of electronics fabrication.

- *Supplementary Methods:*

Theoretical estimation of shape detection performance through the two-step transmission process

Theoretical estimation and experimental validation of the negligible depth effect on shape sensing

SUPPLEMENTARY FIGURES

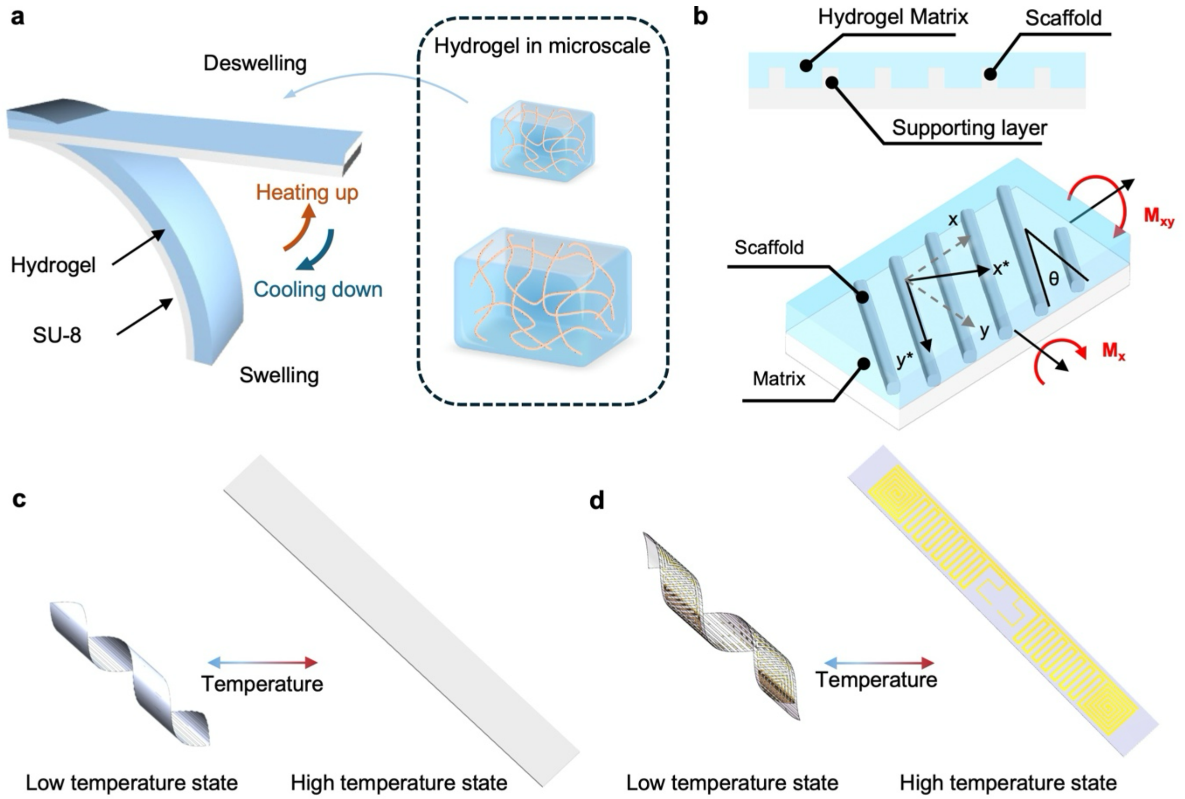

Supplementary Fig. 1. Mechanism of helical structure formation via the interplay of hydrogel swelling and anisotropic supporting layer. a. Illustration of the microscale swelling and deswelling mechanism in pNIPAM hydrogel, leading to the bending of hydrogel/SU-8 bilayer structures. The hydrogel matrix, a polymer crosslinked network, absorbs water molecules, causing it to bend around the easy axis below the lower critical solution temperature (LCST). When the temperature exceeds the LCST, the hydrogel becomes more hydrophobic, expelling water molecules from the matrix, subsequently shrinking in volume and returning to the planar shape. **b.** Mechanism showing that the anisotropic layer converts bending moments into torque, twisting the planar shape into a helical shape upon hydrogel swelling. Schematic of the shape reconfiguration of (**c**) the microrobotic structure and (**d**) the dipole antenna upon temperature changes.

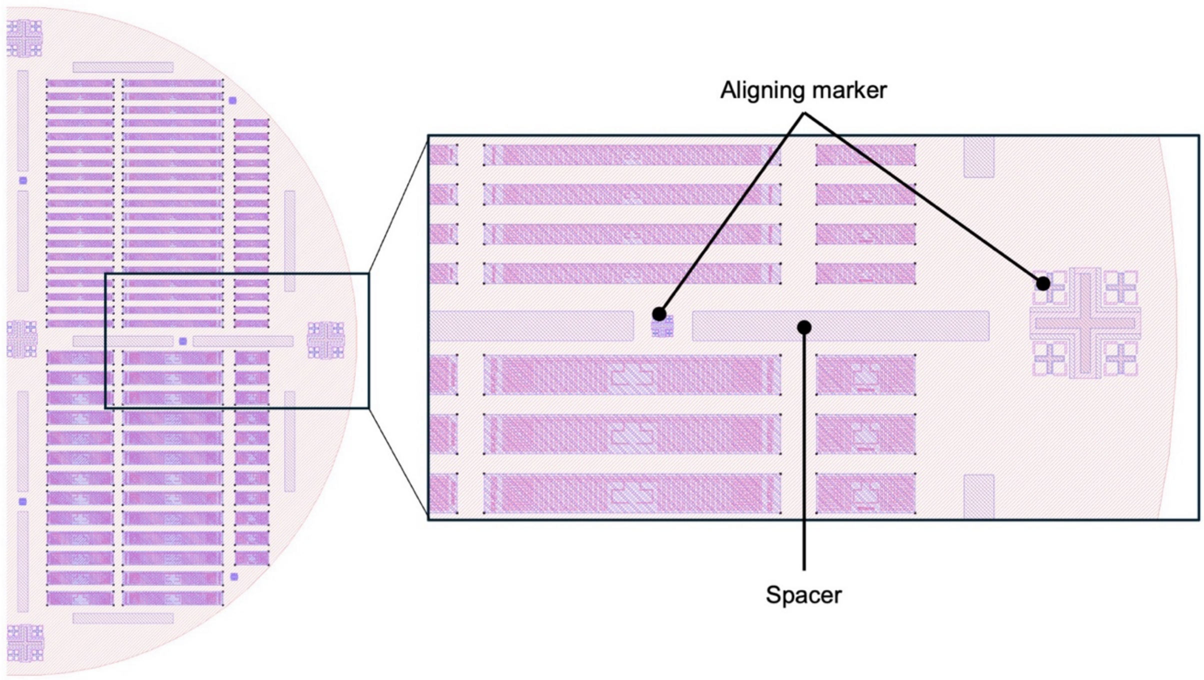

Supplementary Fig. 2. Alignment marker and spacer in mask design for precise layered structure fabrication. This image displays the alignment markers designed in the mask layout, which enhance alignment accuracy during 2-D lithography and hydrogel fabrication. The spacer shown in the image is a crucial component of the sandwich-like fabrication method, enabling precise control over the thickness of the hydrogel.

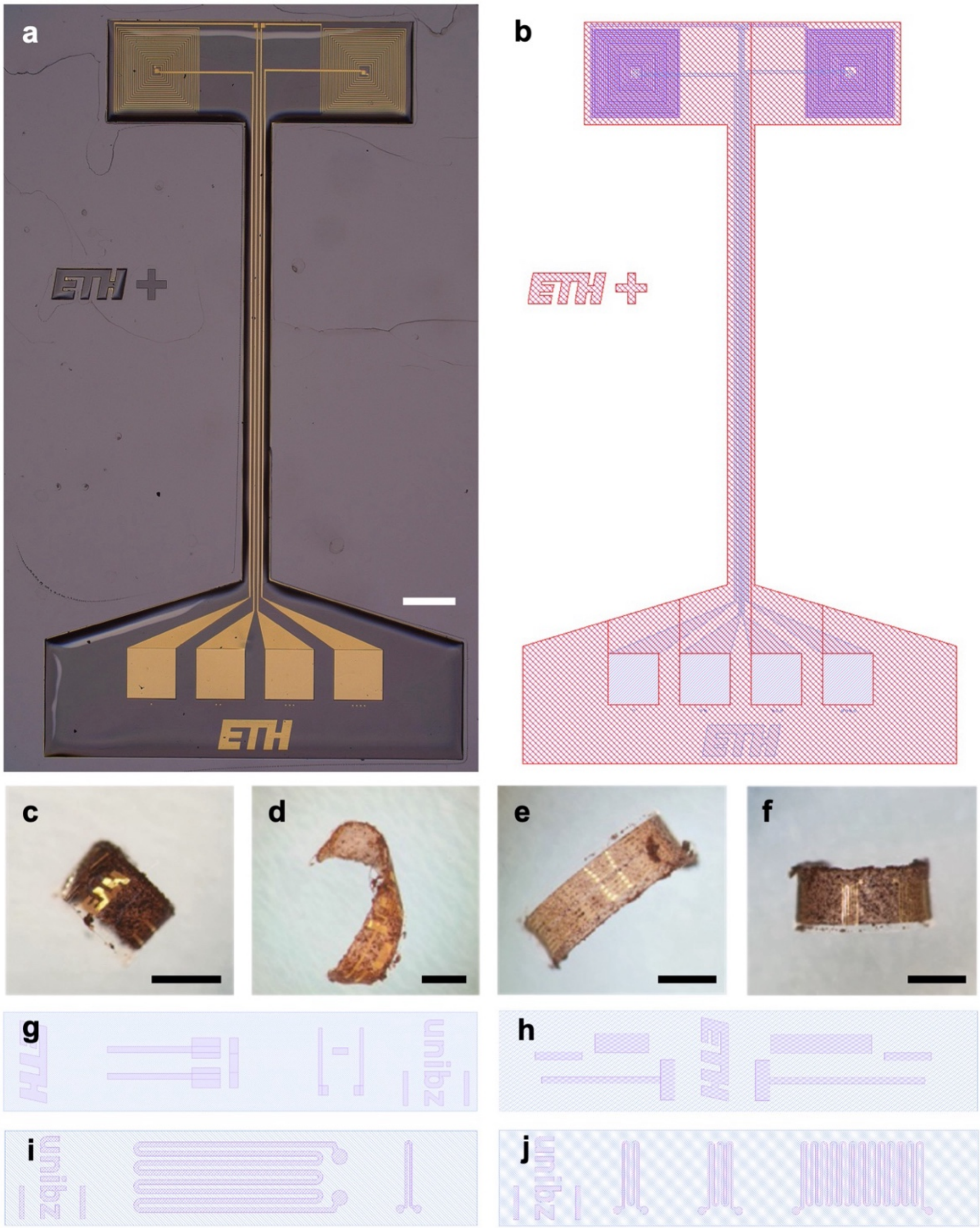

Supplementary Fig. 3. Capability of using various electronics designs in integration. a. An optical image of a tethered multi-coil structure after fabrication. **b**. The layout design of (**a**). **c-f.** Optical images of the integrated microrobots with various patterns of electronics. **g-j**. The layout designs of (**c-f**), respectively. The results in (**a,b**) demonstrate that the electronics and passive layer can be successfully fabricated with the system on chip technology. The results in (**c-f**) indicate that the integration methods are compatible with various electronics. Note that integrated structures without scaffold layer form irregular shape according to the electronic patterns. This general method can integrate functional electronics with soft microrobots (Scale bar: 1 mm).

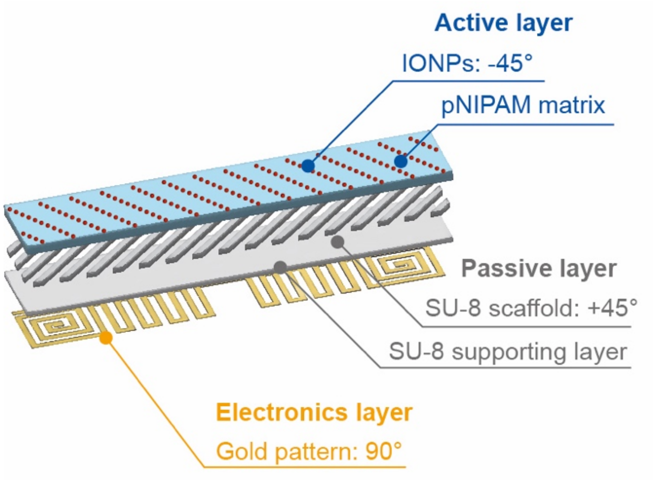

Supplementary Fig. 4. Layer configuration integrating flexible electronics and soft microrobots. A patterned electronics layer is a dipole antenna. While an active layer is composed of iron oxide nanoparticle (IONP)-thermoresponsive hydrogel composites, a passive layer comprises a supporting layer and a scaffold layer.

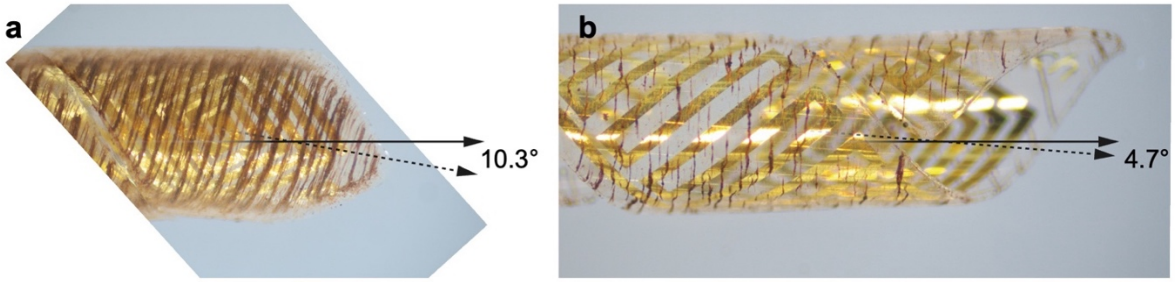

Supplementary Fig. 5. Effect of the IONP concentration ratio on helical shape formation. a. With high IONP concentration ratio. **b.** With low IONP concentration ratio. The angle between the axis perpendicular to the anisotropic scaffold and the helical axis changes from 10.3° with a high concentration of IONPs to 4.7° with a low concentration of IONPs. While IONP concentrations can change the helical angles, the effect is negligible.

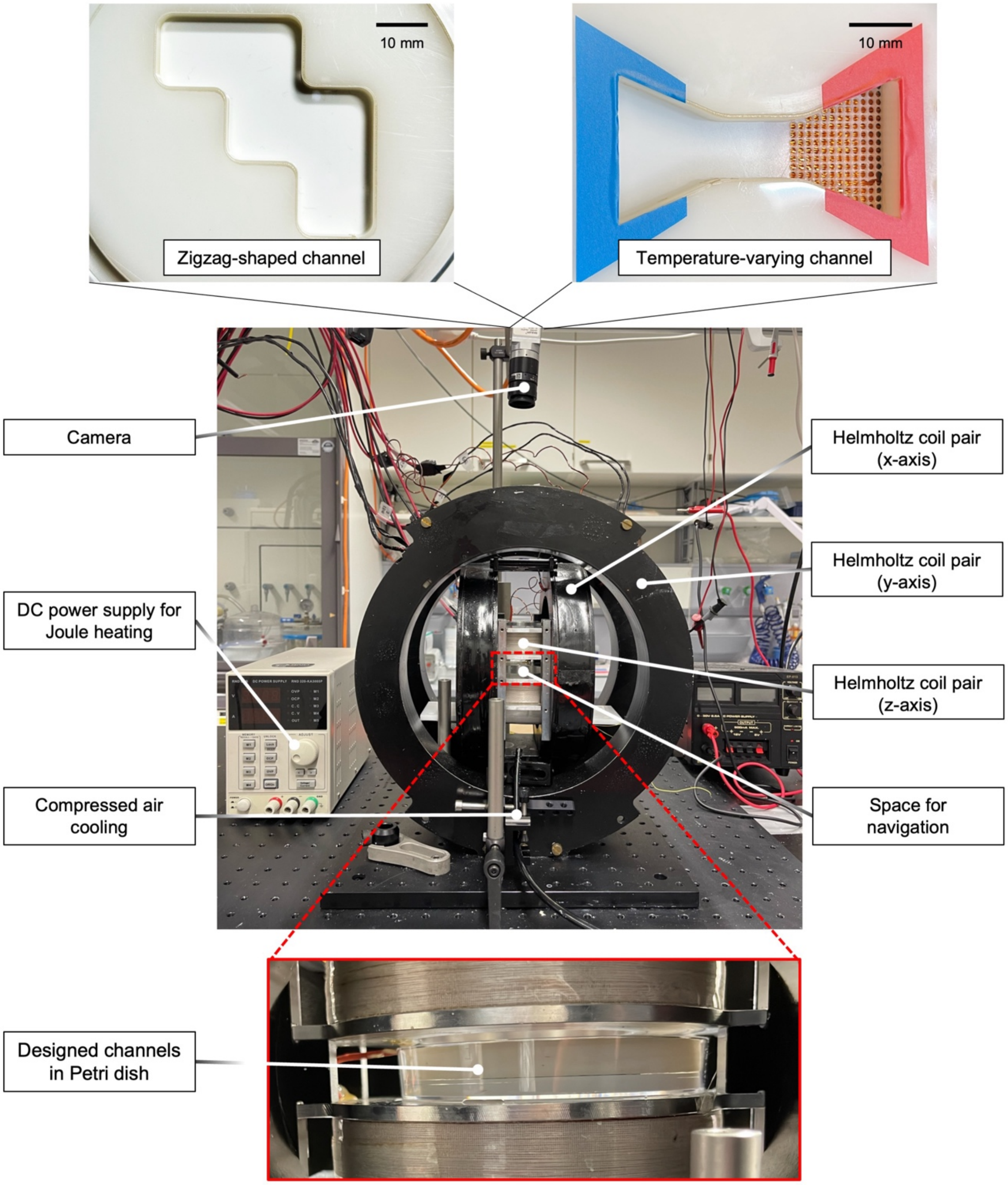

Supplementary Fig. 6. Magnetic navigation setup using three pairs of Helmholtz coils. The system includes three pairs of Helmholtz coils, generating a rotating magnetic field in the x, y, or z axis with a maximum intensity of 50 mT and a frequency of 10 Hz. Two types of channels (zigzag-shaped channel and temperature-varying channel) are placed in the space between the Helmholtz coils. The temperature-varying channel features a Joule heater (right side) positioned beneath the holes at the bottom surface. A DC power supply powers the Joule heater to warm the environment within the channel, facilitating the shape transformation. A camera is mounted on the top for video capturing.

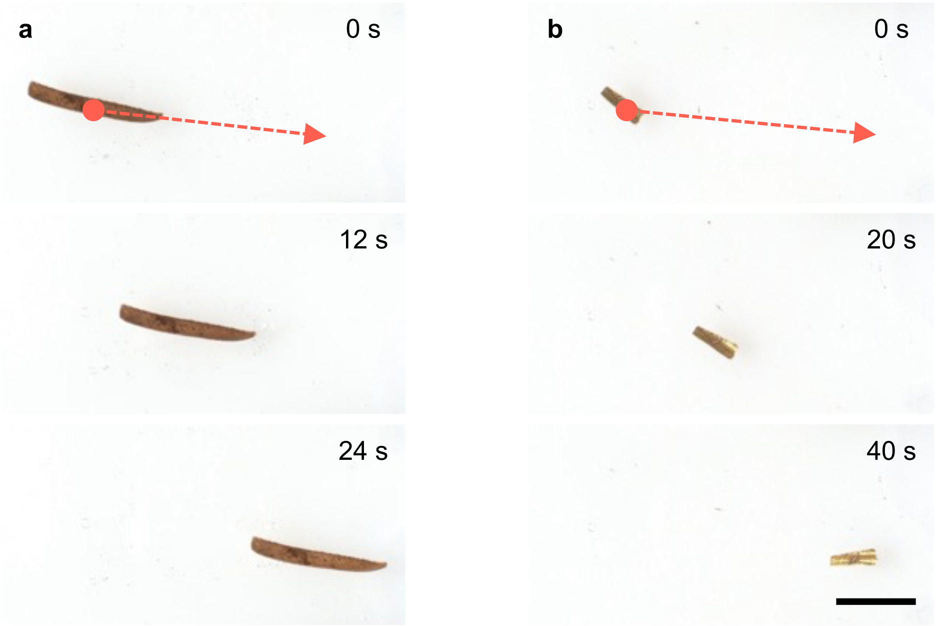

Supplementary Fig. 7. Navigation of planar microrobots using a magnetic gradient field. A 2 mm × 15 mm planar microrobot (**a**) and a 2 mm × 5 mm planar microrobot (**b**) were guided by the magnetic dragging force. The magnetic field and gradient were set to 10 mT and 120 mT mm^{-1}, respectively. The average speeds of 2 mm × 15 mm and 2 mm × 5 mm microrobot were 1.2 mm s^{-1} and 0.7 mm s^{-1}, respectively (Scale bar: 10 mm).

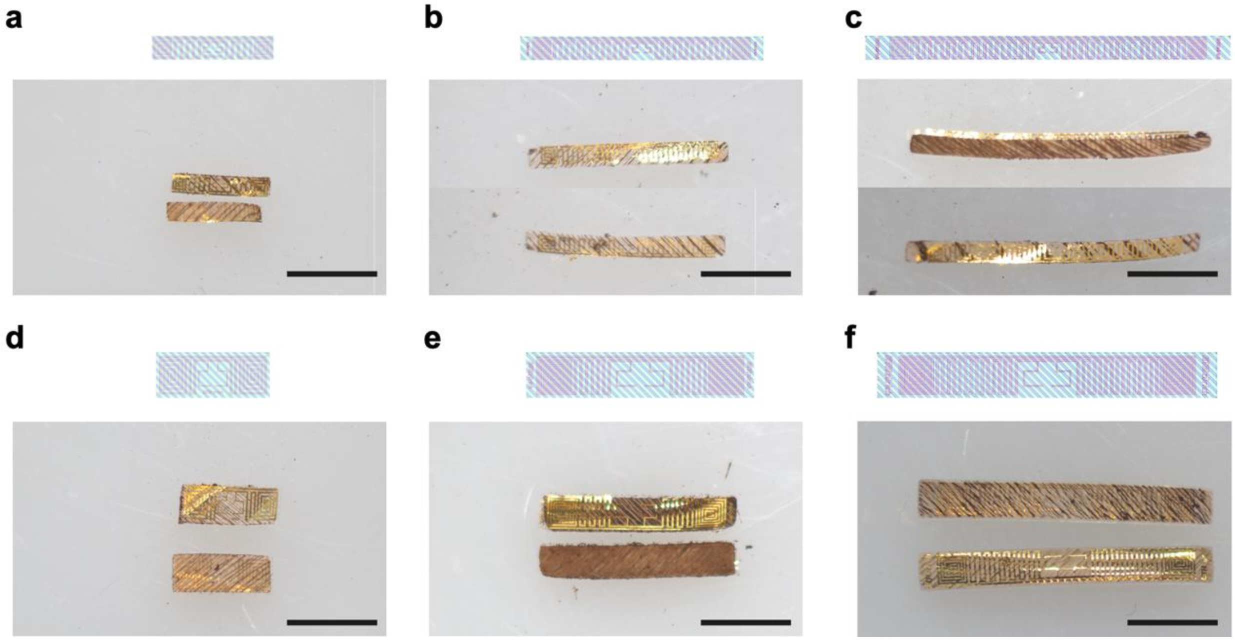

Supplementary Fig. 8. Layout designs and both sides optical images of the microrobot in planar form, shown in various dimensions. a. 1 mm × 5 mm. **b.** 1 mm × 10 mm. **c.** 1 mm × 15 mm. **d.** 2 mm × 5 mm. **e.** 2 mm × 10 mm. **f.** 2 mm × 15 mm. In the layout design, blue and gray colors indicate microrobotic pattern design, while purple represents the electronics pattern. The multilayer structure of the microrobot successfully incorporates the electronics, passive layer, and active layer. Additionally, the IONPs are successfully aligned under the magnetic field (Scale bar: 5 mm).

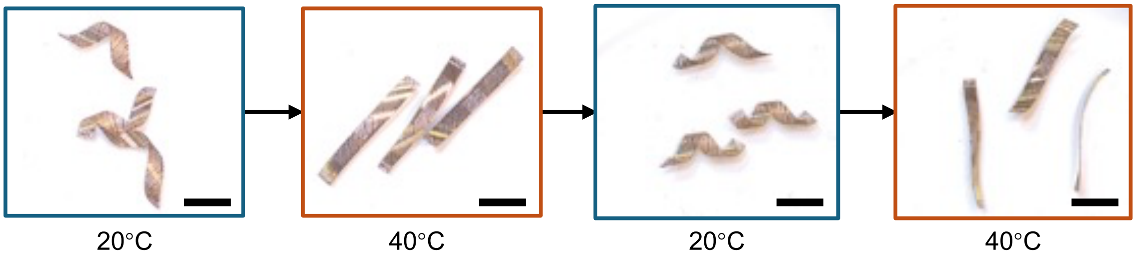

Supplementary Fig. 9. Shape reconfiguration repeatability test. This repeated test confirms the reliable and repeatable shape reconfiguration capability of the microrobots (Scale bar: 5 mm).

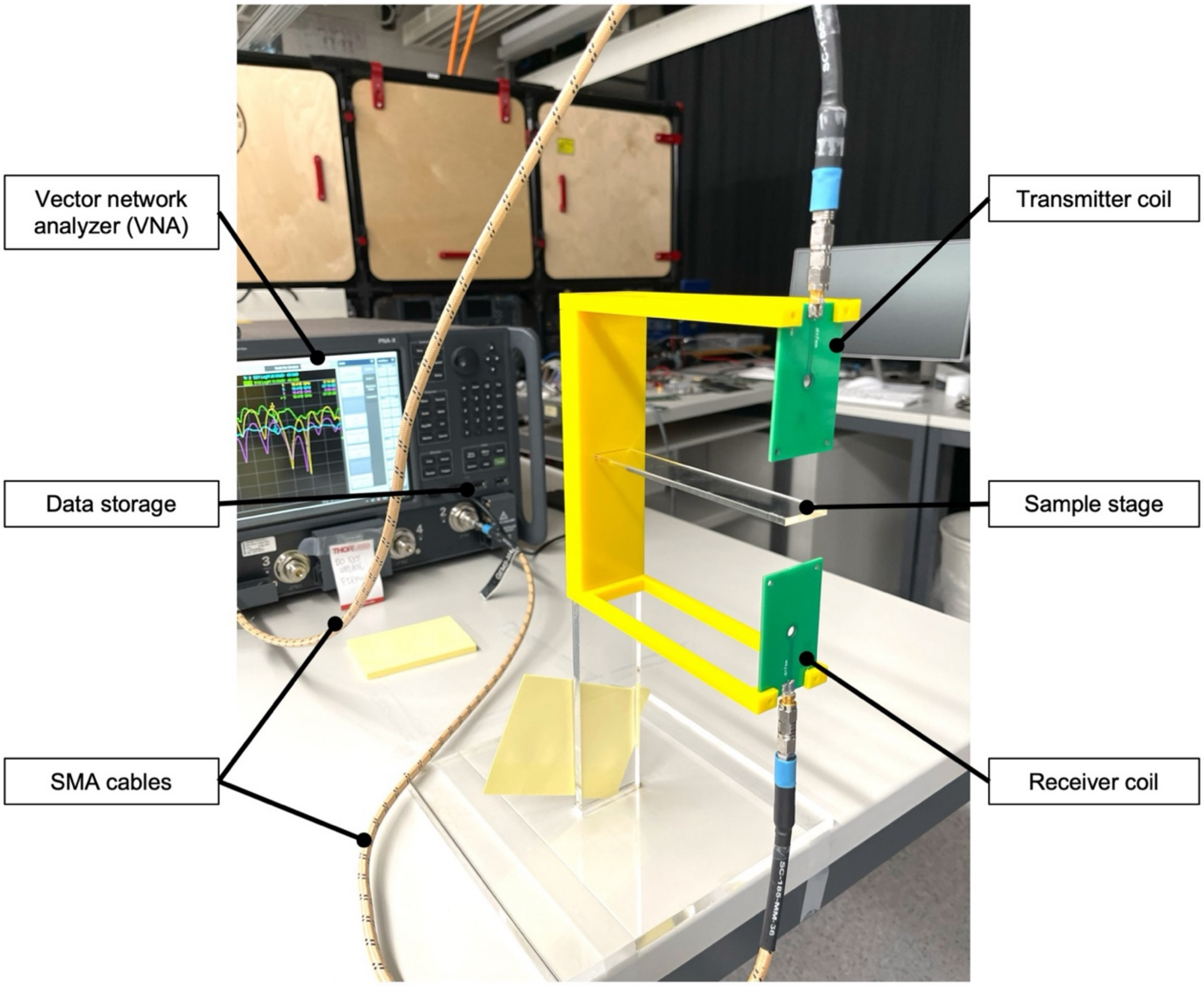

Supplementary Fig. 10. Experimental setup for evaluating shape detection performance through the radio communication. The transmitter and receiver coils are mounted on a 3-D printed holder, with the acrylic sample stage placed between the two coils. The coils are connected to a vector network analyzer (VNA, KEYSIGHT© N5247B PNA-X Microwave Network Analyzer, 10 MHz to 67 GHz) via SMA cables. The transmission coefficient, S_{21}, is measured for further analysis.

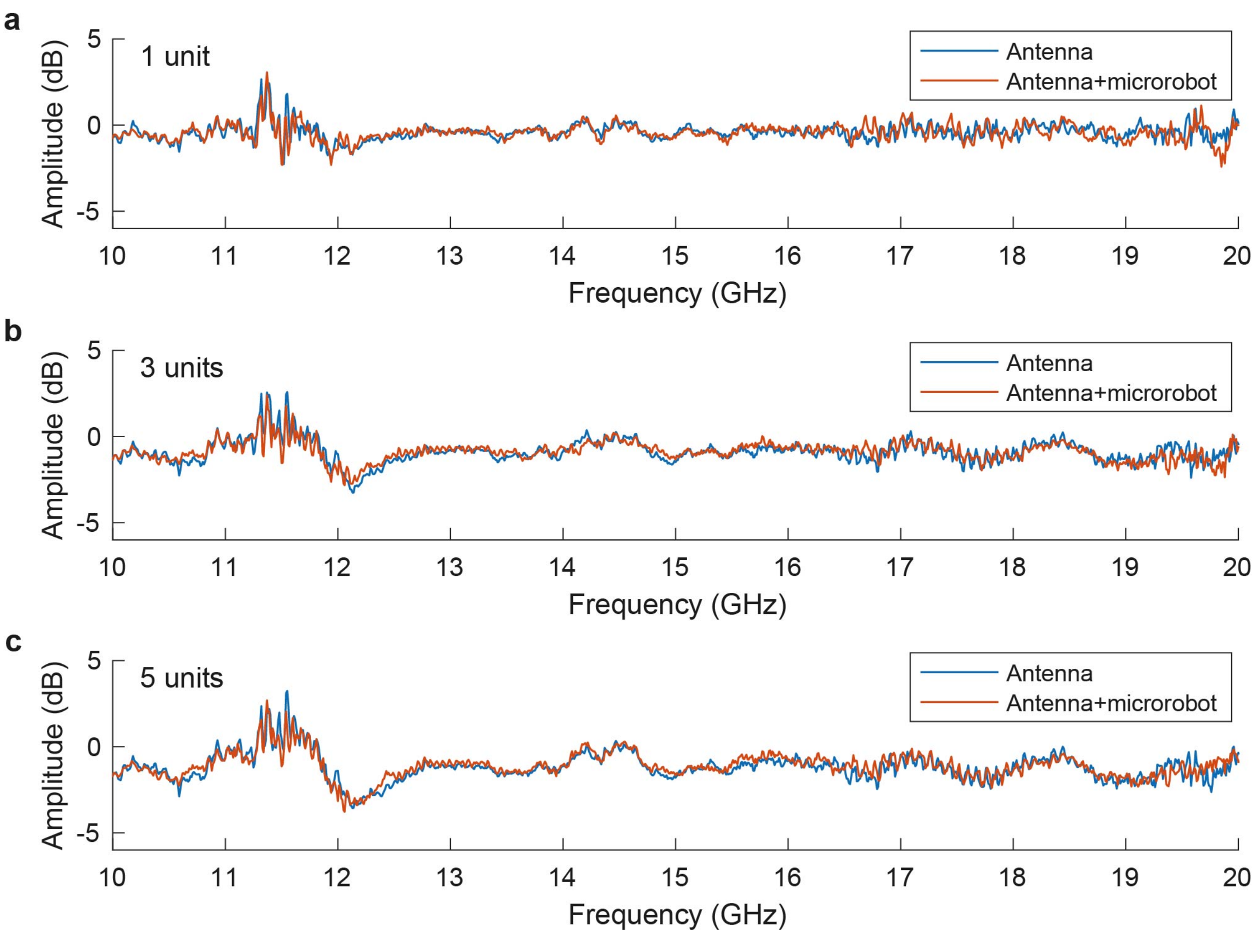

Supplementary Fig. 11. Negligible effect of IONPs and hydrogel on radio communication. Signals from (**a**) one microrobot, (**b**) three microrobots, and (**c**) five microrobots. 'Antenna' indicates standalone electronics, while 'Antenna+Robot' indicates the integrated electronics with microrobots. Both individual and collective configurations of microrobots and antennas were tested under identical conditions. The RF signal showed no significant difference between the antennas integrated with the microrobots and the standalone antennas.

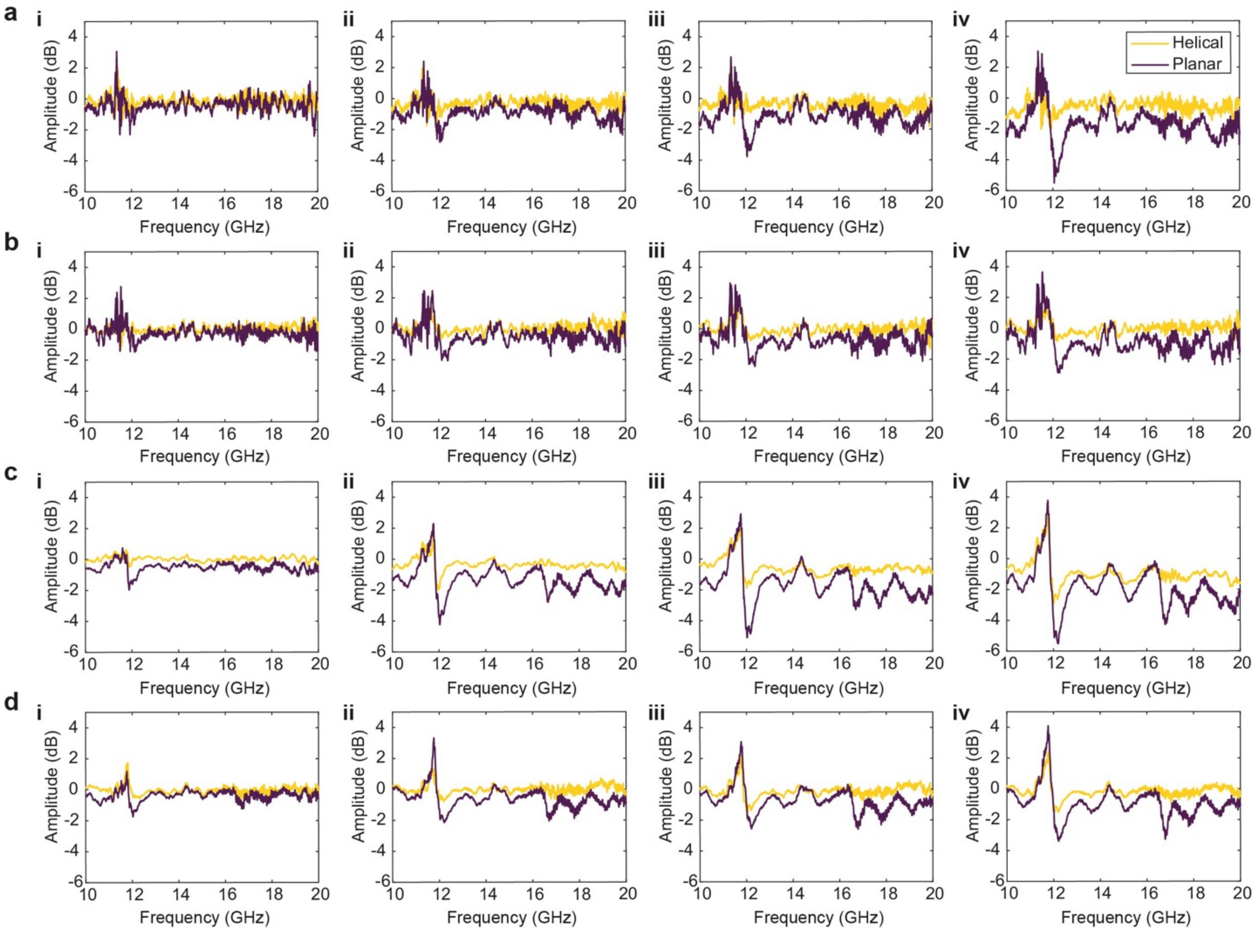

Supplementary Fig. 12. Collective effect of microrobots with different dimensions on radio communication. a. Distance: 10 cm, Microrobot: 2 mm × 15 mm. **b.** Distance: 10 cm, Microrobot: 2 mm × 10 mm. **c.** Distance: 5 cm, Microrobot: 2 mm × 15 mm. **d.** Distance: 5 cm, Microrobot: 2 mm × 10 mm. In each column, (**i**) to (**iv**) represent experiments with one microrobot, three microrobots, five microrobots, and seven microrobots, respectively. The radio communication signal difference between shapes becomes more distinct with an increasing number of microrobots, demonstrating the collective effect with both 2 mm × 15 mm and 2 mm × 10 mm microrobots. Note that panel (**a**) is duplicated from the main for reference.

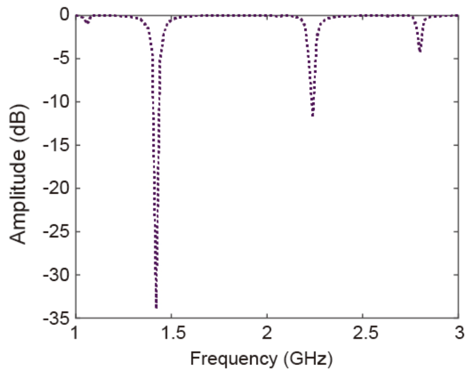

Supplementary Fig. 13. Simulation of medium effects on RF communication. The COMSOL simulation with a PBS medium shows a resonant frequency shift to a lower region, consistent with experimental results.

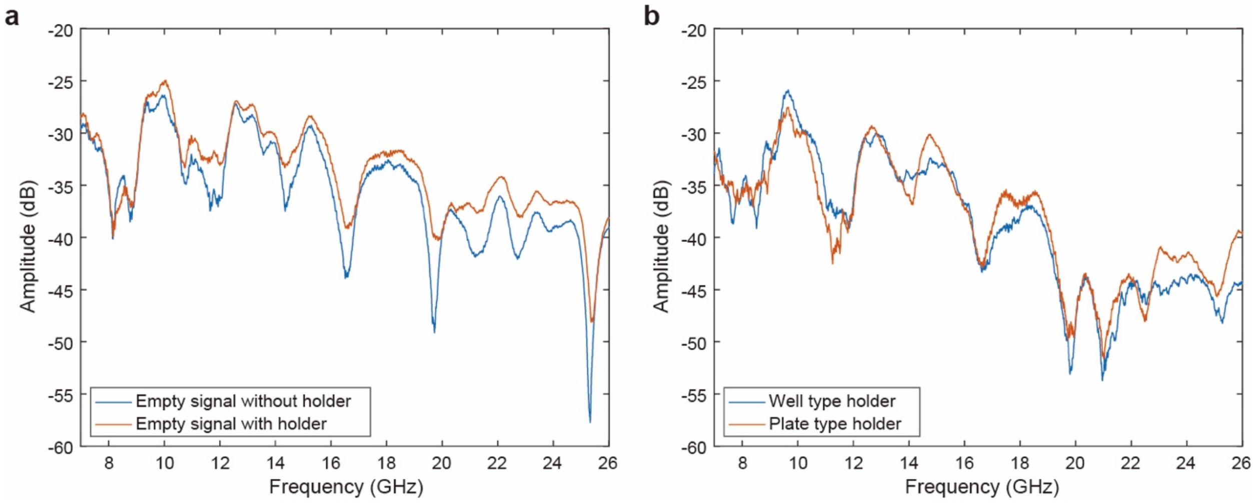

Supplementary Fig. 14. Effect of the sample holder on radio communication. a. RF communication signals recorded with and without the holder. **b.** RF communication signals recorded using a well-type holder and a plate-type holder. While the overall trends remain similar across different conditions, slight amplitude shifts are observed. These signals are superimposed on background noise and post-processed to isolate the environmental effect.

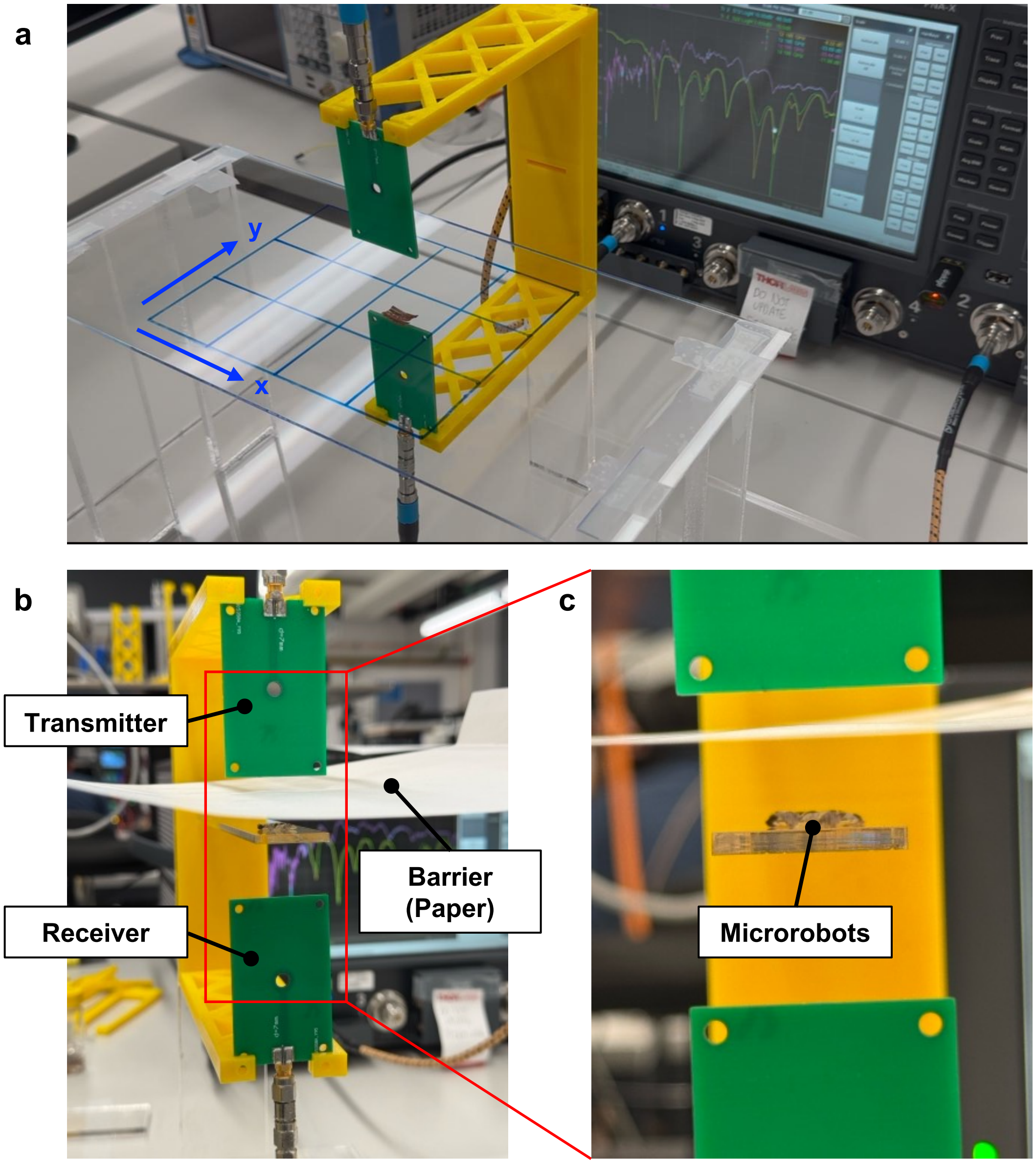

Supplementary Fig. 15. Localization setup and shielded experimental setup. a. The experimental platform, divided into a 4 × 3 array. Two transmission coils were connected to the VNA, and the microrobots were positioned at (3,2). **b.** A shielded paper cover was placed between the microrobots and the transmitter coil, with an enlarged view shown in **(c)**.

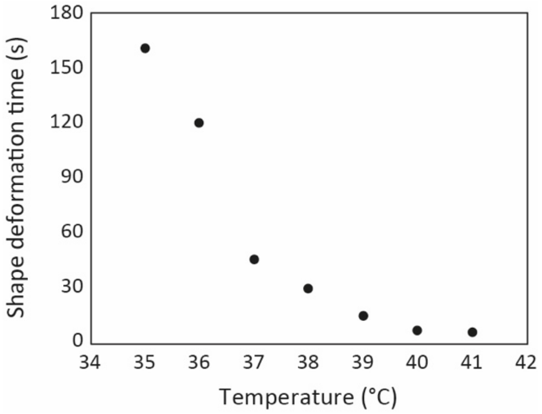

Supplementary Fig. 16. Time for shape deformation from helical to planar as a function of temperature. Note that the exact deformation time may vary depending on factors such as magnetic nanoparticle density, crosslinking density, and film thickness. However, the overall trend remains consistent.

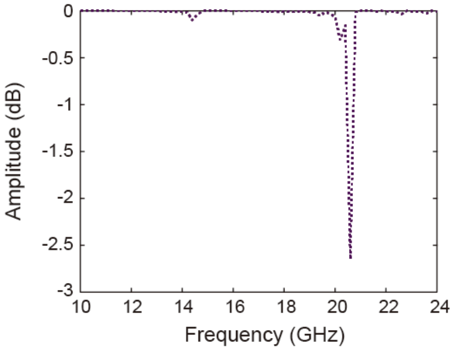

Supplementary Fig. 17. Resonant frequency of the new spiral antenna in air. The resonant frequency of the spiral antenna is ~ 20.6 GHz.

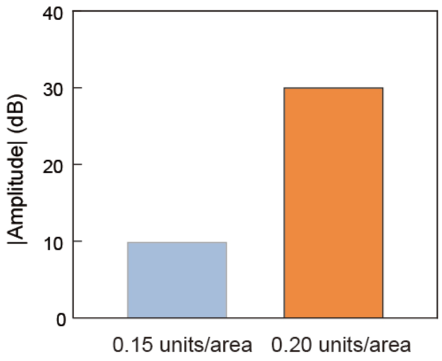

Supplementary Fig. 18. Effect of microrobot distribution density on RF communication using COMSOL simulation. A 33% increase in microrobot distribution density resulted in a threefold enhancement of the S_{21} amplitude at the resonant frequency (Unit area: 1 cm^2).

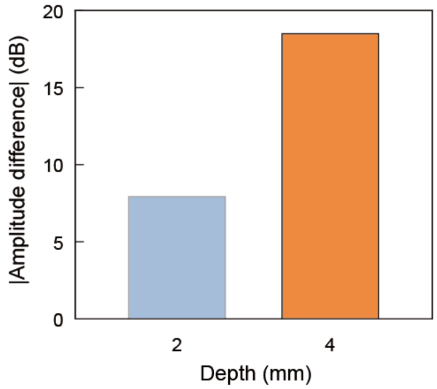

Supplementary Fig. 19. Experimental comparison of microrobots' RF signal response at the resonant frequency in PBS. As the depth increases, communication performance improves, leading to a more significant absolute amplitude difference.

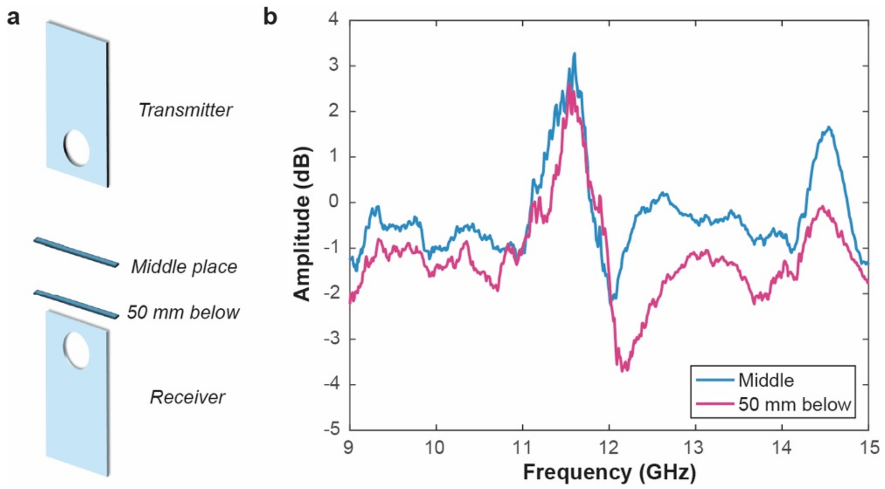

Supplementary Fig. 20. Experimental comparison of seven microrobots' RF signal response at two different depths. a. Schematic diagram of experimental geometry with two different antenna depths. **b.** As the depth increases, although attenuation affects amplitude, spectrum remains clearly distinguishable.

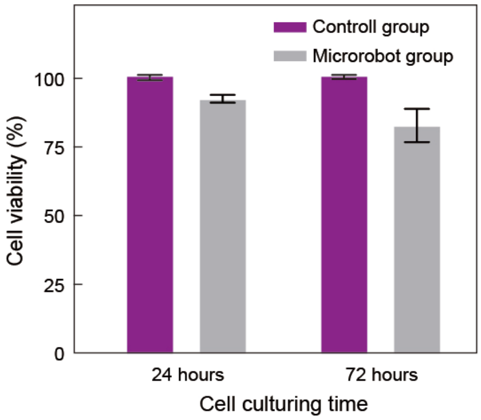

Supplementary Fig. 21. Biocompatibility of microrobots. Using MTT assays with human umbilical vein endothelial cells (HUVEC), the microrobots were subjected to cell culturing conditions for 24 and 72 hours, demonstrating sufficient biocompatibility. Error bars represent standard deviation.

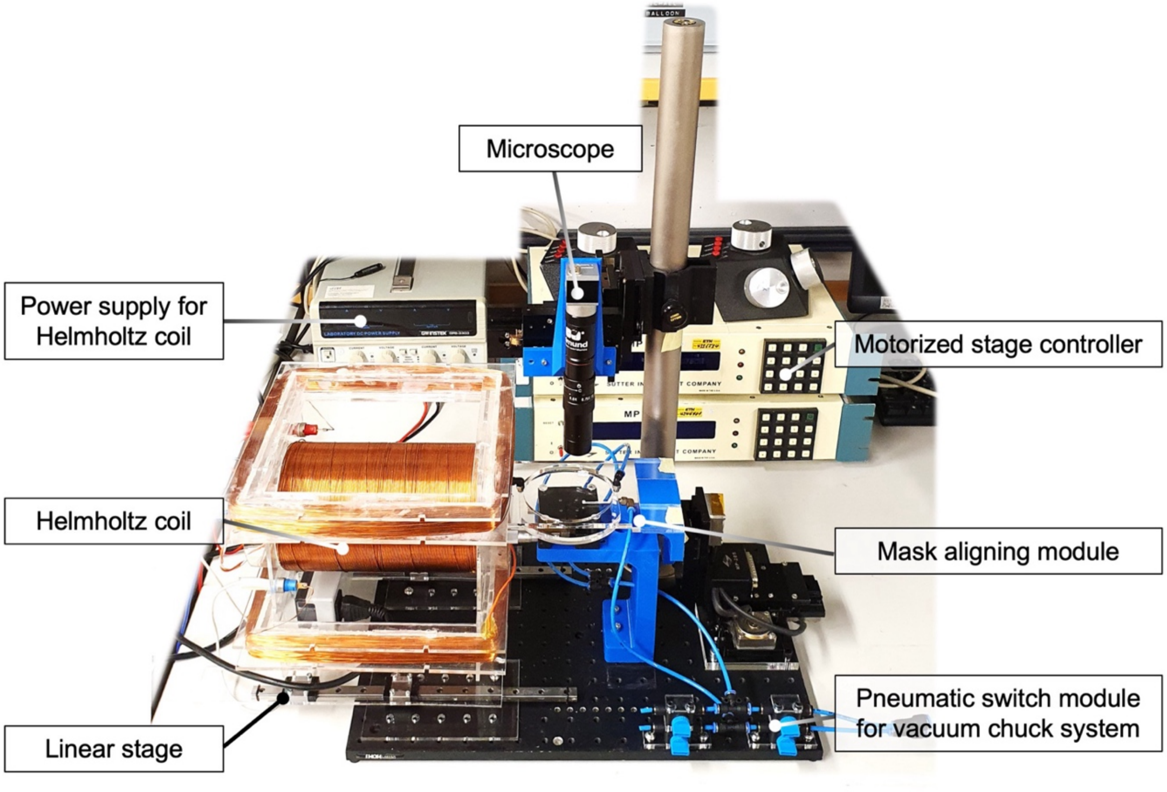

Supplementary Fig. 22. Active layer fabrication setup. The glass photomask and silicon wafer (with electronics and passive layer on top) were positioned on a mask-aligning module and held in place by vacuum chucks. The photomask and wafer were aligned using a microscope, motorized 3-axis stages, and a mechanical rotating stage. Once aligned, the hydrogel resin was injected into the space between the photomask and wafer and pressed down by lowering the motorized stage. Subsequently, the microscope was removed, and the Helmholtz coil was shifted into the center to align the IONPs. A UV lamp within the coil was then activated to initiate the crosslinking of the hydrogel resin.

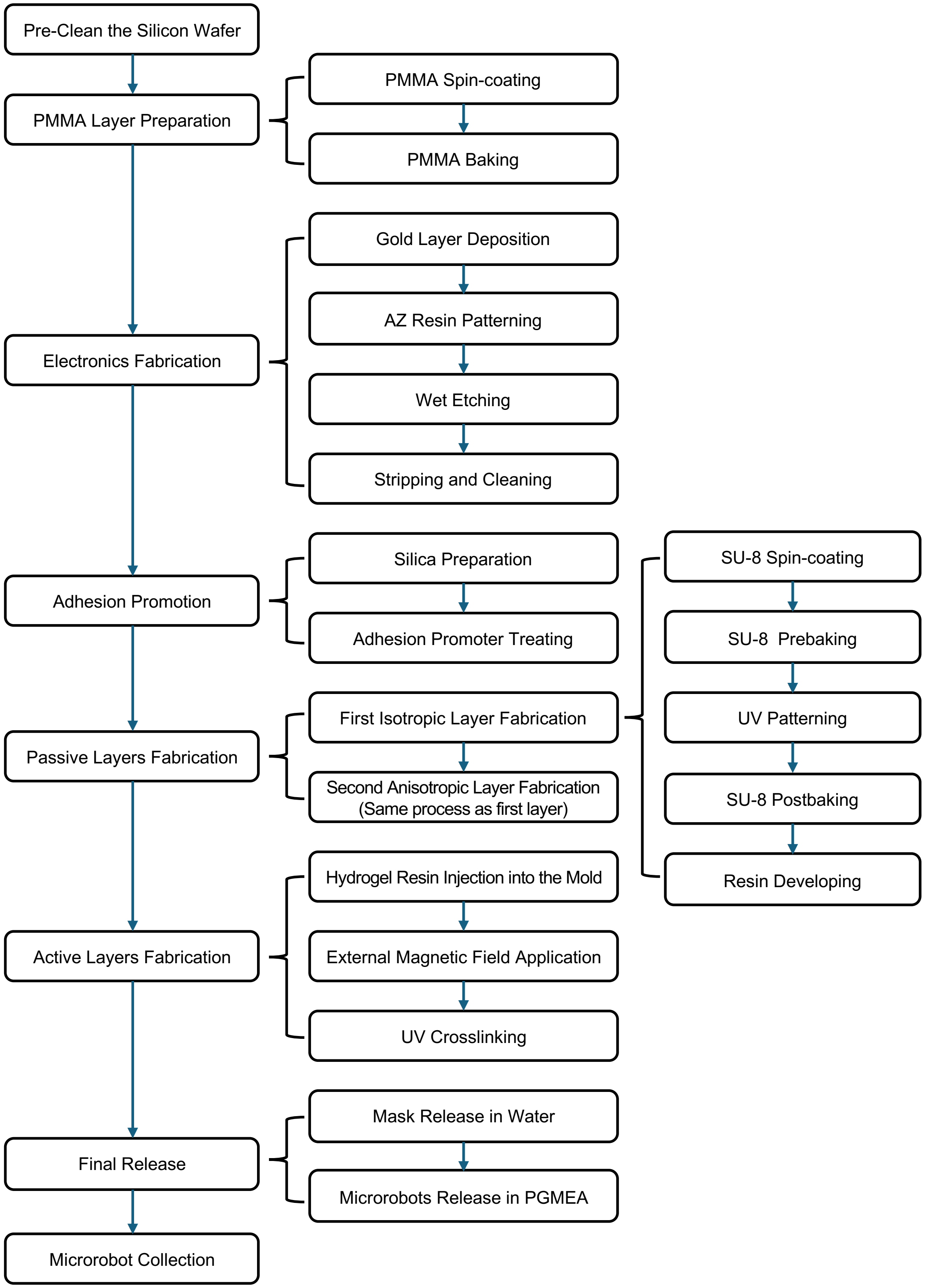

Supplementary Fig. 23. Fabrication and integration workflow chart.

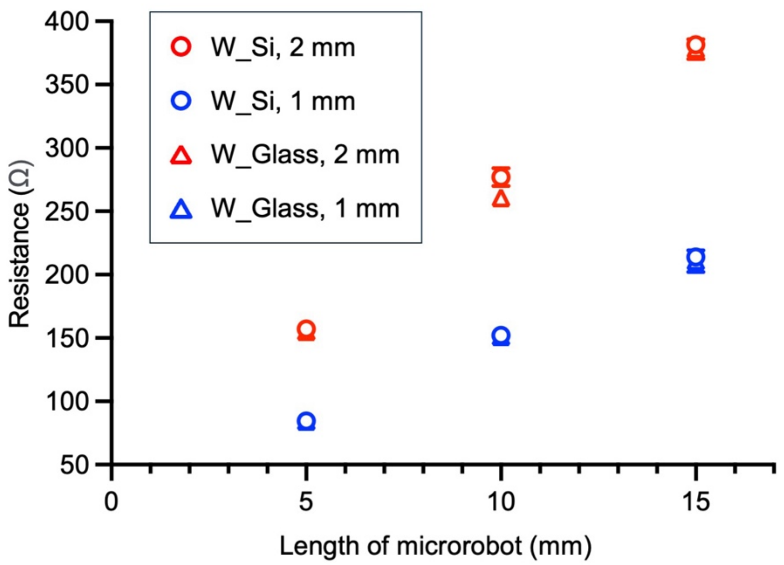

Supplementary Fig. 24. Stability of electronics fabrication. We measured the resistance of antenna patterns with varying dimensions using a probe station to assess the stability of the electronics fabrication process. The results demonstrate an almost linear relationship with length increase, aligning with theoretical predictions from antenna design parameters. Furthermore, the same antenna patterns on different substrates showed no significant differences. W_Si and W_Glass refer to the width of the microrobots fabricated on silicon and glass wafers, respectively. Error bars represent standard deviation.

SUPPLEMENTARY METHODS

Theoretical estimation of shape detection performance through the two-step transmission process

Shape detection is performed through the radio communication signal of a dipole antenna, which is placed between a transmitter coil and a receiver coil. This radio communication involves a two-step transmission process. The first step is the transmission between the transmitter coil and the dipole antenna, which acts as the receiver. The second step is the transmission between the dipole antenna, now serving as the transmitter, and the receiver coil. These steps adhere to the transmission theory of antennas[41].

The general Friis transmission equation is given by:

$$P_{\mathrm{r}} = P_{\mathrm{t}} G_{\mathrm{t}} G_{\mathrm{r}} \left(\frac{\lambda}{4\pi R}\right)^2 \tag{9}$$

where P_r and P_t are the power received by the receiver and the power transmitted from the transmitter, respectively. G_t and G_r are the gains of the transmitter and receiver, respectively. λ is the wavelength, and R is the distance between the transmitter and receiver.

Thus, for the first step, the power received by the dipole antenna is expressed as:

$$P_{\mathrm{r,dipole}} = P_{\mathrm{t}} G_{\mathrm{t}} G_{\mathrm{r,dipole}} \left(\frac{\lambda}{4\pi R_1}\right)^2 \tag{10}$$

where $P_{r,dipole}$ and $G_{r,dipole}$ are the received power and gain of the dipole antenna, respectively. R_1 is the distance between the transmitter coil and the dipole antenna. Similarly, for the second step, the power received by the receiver coil is expressed as:

$$P_{\mathrm{r}} = P_{\mathrm{t,dipole}} G_{\mathrm{t,dipole}} G_{\mathrm{r}} \left(\frac{\lambda}{4\pi R_2}\right)^2 \tag{11}$$

where $P_{t,dipole}$ and $G_{t,dipole}$ are the received power and gain of the dipole antenna, respectively. R_2 is the distance between the receiver coil and dipole antenna.

During the re-radiation process, the power transmitted out by the dipole antenna can be expressed as:

$$P_{\mathrm{t,dipole}} = P_{\mathrm{r,dipole}} \cdot \Gamma^2 \quad (12)$$

where Γ is a constant to describe the reflection coefficient of the dipole antenna, which is 1 in our antenna.

Thus, using Equation (10) and (12), Equation (11) can be expressed as:

$$P_{\mathrm{r}} = P_{\mathrm{t}} G_{\mathrm{t}} G_{\mathrm{r}} \left(\frac{\lambda}{4\pi R_1}\right)^2 \left(\frac{\lambda}{4\pi R_2}\right)^2 G_{\mathrm{r,dipole}} G_{\mathrm{t,dipole}} \quad (13)$$

Here, P_t, G_t, G_r, R_1, R_2, and λ are constant within the experimental configuration. Given that $G_{r,dipole}$ and $G_{t,dipole}$ are related with the maximum directivity D_0, we also get:

$$G_{\mathrm{r,dipole}} = G_{\mathrm{t,dipole}} \approx \eta D_0 \quad (14)$$

where η is the radiation efficiency of the antenna. Thus, Equation (13) can be reformulated as:

$$P_{\mathrm{r}} = P_{\mathrm{t}} G_{\mathrm{t}} G_{\mathrm{r}} \left(\frac{\lambda}{4\pi R_1}\right)^2 \left(\frac{\lambda}{4\pi R_2}\right)^2 \eta^2 {D_0}^2 \quad (15)$$

Finally, the transmission coefficient is described as:

$$|S_{21}|^2 = \frac{P_{\mathrm{r}}}{P_{\mathrm{t}}} = \eta^2 G_{\mathrm{t}} G_{\mathrm{r}} \left(\frac{\lambda}{4\pi R_1}\right)^2 \left(\frac{\lambda}{4\pi R_2}\right)^2 {D_0}^2 \quad (16)$$

where the maximum directivity D_0 of the dipole antenna can be expressed as:

$$D_0 = \frac{4\pi}{\lambda^2} A_{\mathrm{em}} \quad (17)$$

where A_{em} is the effective aperture of the dipole antenna. Consequently, the shape transformation of microrobots from a helical to a flat shape increase the effective aperture of the dipole antenna (A_{em}), leading to $D_{0,planar} > D_{0,helical}$ and a different transmission coefficient (S_{21}) in response to the shape.

Theoretical estimation and experimental validation of the negligible depth effect on shape sensing

The remote communication system follows a two-hop relay architecture, with an antenna-embedded microrobot serving as the intermediate node between the transmitter and receiver. The transmission coefficient S_{21} is derived from Equation (16) as:

$$|S_{21}| = \sqrt{\frac{P_r}{P_t}} = \eta D_0 \sqrt{G_\mathrm{t} G_\mathrm{r}} \left(\frac{\lambda}{4\pi}\right)^2 \left(\frac{1}{R_1 R_2}\right). \quad (18)$$

In this system, the transmitter and receiver positions are fixed, with the distance between the two coils defined as D. When the antenna position varies, the parameters η, D_0, G_t, G_r, and λ remain constant. The change in antenna position along the depth direction, denoted Δ, alters the distances between the microrobot and the transmitter (R_1) and receiver (R_2), described by:

Before movement,

$$R_1 = R_2 = \frac{D}{2}. \quad (19)$$

After movement,

$$R_1 = \frac{D}{2} + \Delta, \qquad R_2 = \frac{D}{2} - \Delta. \quad (20)$$

In this case, the transmission coefficient S_{21} depends solely on the $(R_1R_2)^{-1}$. The path loss due to depth variation, $L(\Delta)$, is given by:

$$L(\Delta) = -20\log(R_1 R_2). \quad (21)$$

Applying a Taylor expansion yields an approximate expression:

$$L(\Delta) \approx -\frac{20\Delta^2}{\mathrm{In}10\left(\frac{D}{2}\right)^2} = -34.74\left(\frac{\Delta}{D}\right)^2. \quad (22)$$

To validate the theoretical analysis, we conducted experiments to measure the S_{21} signal variation at two different antenna locations. In the first configuration, seven planar antennas were positioned at the midpoint between the transmitter and receiver (D = 120 mm). In the second, the same antennas were shifted 50 mm

toward the receiver. The measured path loss between these two configurations was approximately −1.5 dB, which agrees well with the theoretical prediction based on Equation (22). Overall, the signal variation due to depth is roughly half the magnitude of that caused by morphological changes in air, which can reach to −4 dB (Supplementary Fig. 20). Notably, despite this attenuation, the resonance valley in the spectrum remains clearly distinguishable, indicating that RF-based shape sensing remains feasible under moderate depth variation.
