# Supplementary material for: Soft magnetic microrobots with remote sensing and communication capabilities
Source: Nat Commun. 2025 Nov 25;16:10489. doi: 10.1038/s41467-025-65459-8 (PMC12647796; doi:10.1038/s41467-025-65459-8)
Supplement: Supplementary file 2 — Description of Additional Supplementary Files [file 41467_2025_65459_MOESM2_ESM.pdf]

Description of Additional Supplementary Files

Supplementary Movie 1. Shape morphing simulation of planar hydrogel/SU-8 bilayer structures.

Supplementary Movie 2. Surface treatment effect preventing delamination during shape transformation.

Supplementary Movie 3. Magnetic locomotion using rotating magnetic fields: demonstrations in open space

and zigzag-shaped channel navigation.

Supplementary Movie 4. Magnetic locomotion using gradient magnetic fields: navigation of planar

microrobots.

Supplementary Movie 5. Repeatability of shape transformation.

Supplementary Movie 6. Magnetic navigation and thermoresponsive shape transformation in temperature varying channel
